# Estimation of the burden of disease averted by leisure center membership across Spain

**DOI:** 10.3389/fspor.2025.1575583

**Published:** 2025-09-05

**Authors:** Alfonso Jimenez, Inés Nieto, Xian Mayo, Pablo Bascones, Jesús De Soto Cardenal, Alfonso Arroyo, Gary Liguori, Larissa Davies

**Affiliations:** ^1^ Research Centre in Sports Science, King Juan Carlos University, Fuenlabrada, Madrid, Spain; ^2^ GO fit LAB, GO fit Life, Science and Technology, S.A. Alcobendas, Madrid, Spain; ^3^ Sustainability and Climate Change, PricewaterhouseCoopers (PwC), Madrid, Spain; ^4^ Penn State Abington, Abington, PA, United States; ^5^ Sport Policy Unit, Department for People and Performance, Faculty of Business and Law Institute of Sport, Manchester Metropolitan University, Manchester, United Kingdom

**Keywords:** physical activity, burden of disease, leisure centers, mortality, DALY

## Abstract

**Introduction:**

Active behavior performed in leisure centers might help reduce the negative health impacts associated with physical inactivity. The disability-adjusted life years (DALYs) is a valuation technique to quantify lifetime disease burden including both non-fatal health consequences of diseases and premature death.

**Method:**

This study estimated the role of the largest leisure center in Spain (GO fit) in averting the burden of five diseases and premature deaths during 2017 as a consequence of the physical activity and exercise programs and services delivered. A preferred model was implemented with a static picture of the burden of disease, without including discounting rate and age-weights. Sensitivity analyses were conducted considering these two variables.

**Results:**

The estimation was that GO fit services could have averted a total of 1,165.74 DALYs (10.96 DALYs per 1,000 members) coming from type 2 diabetes (22.62 DALYs), colorectal cancer (81.16 DALYs), breast cancer (48.72 DALYs), stroke (206.15 DALYs), and coronary heart disease (807.10 DALYs).

**Discussion:**

These results indicate that programs and services delivered in physical activity leisure centers could help the public health agenda aim of promoting a more active lifestyle and reducing the burden of disease associated with physical inactivity.

## 1 Introduction

Despite evidence that physical inactivity (PIA) is a risk factor for disease and mortality (1), 27.5% of adults globally do not meet the recommended level of physical activity (PA) (2). In Spain, 2% of deaths can be attributed to low PA (3), resulting in a yearly healthcare spending of more than 2.3 billion International Dollar (Int$), considering both direct and indirect costs (4). Despite efforts at reducing PIA, policy development in Spain has not yet yielded a reduction in PIA prevalence, therefore continuing to place a significant social and economic burden on Spanish society (5).

Leisure-time PA is the preferred domain to engage in a more active lifestyle by the general population (6) and 30% of the European Union population are members of a club where they engage in sport or recreational PA (7). From a public health perspective, promoting PA through leisure centers might be a good option, since tackling inactive behavior from a personal, social, and environmental perspective at the same time (i.e., an ecological model) might have a greater impact than other interventions more focused on the individual (8). Adult long-term fitness center members highlight accessibility, safety, and comfort as some of the main reasons to choose this way of exercising, which are factors also related to the probability of being active (9, 10). In fact, leisure center members have a greater chance of being active and complying with the World Health Organization (WHO) recommendations for PA, compared with those not using leisure centers (11–13), and they also show a myriad of healthy behaviors that might alleviate the provision of health services in the long run (14). Taking all this into account, leisure centers add value from a public health perspective and can promote a smarter resource allocation.

Considering PIA from a public health perspective, there is robust evidence showing that active behavior helps maintain a good health status, prevent a plethora of chronic diseases, and increase life expectancy (15). Disability-adjusted life years (DALYs) is a valuation technique to quantify lifetime disease burden including both non-fatal health consequences of diseases and premature death (16). In Spain, conservative calculations show that PIA accounted for around 1,393,000 DALYs in 2017 (4). Therefore, the effect that a leisure center operator might have on the Spanish population can be quantified by considering the DALYs averted from engaging in an active behavior as a consequence of day-by-day delivery of services.

As of 2023, GO fit is the largest leisure center operator in Spain with 18 facilities and 198,000 members. GO fit is based on a mixed funding model in which the land is provided by the city council for about 50 years, while GO fit provides the investment to build the facility and deliver the services at a fixed public price. The land is usually tertiary use, so while the city council might not obtain any direct economical or societal benefit of the land, a private company such as GO fit is able to offer positive services reducing the prevalence of PIA by promoting active living. This model allows private-sector initiatives to contribute to the public health agenda, tackling PIA and thus the burden of disease, while alleviating the burden on public-sector budgets (17).

The aim of this study was to estimate the impact of GO fit on averting the burden of disease in its members as a consequence of delivering PA and exercise programs and services. Specifically, this study calculated an estimation of the prevention of DALYs that GO fit could have in 2017.

## 2 Methods

This study was guided by the Global Burden of Disease (GBD) theoretical framework (18, 19), which provides a structured approach to estimate the population-level health effects of activities such as the one evaluated in this research. Specifically, a comparative risk assessment model was used to estimate the averted burden of disease attributable to physical activity, expressed in DALYs. This approach integrates epidemiological evidence on the relationship between physical inactivity and disease incidence with population-level exposure data, allowing for the quantification of health gains associated with increased physical activity in leisure center settings.

### 2.1 Participants

The total number of adult members (*n* = 169,031) and the distribution of members based on gender and age were derived from the 2017 annual report of the company (20) (Table 1)*.* An individual was considered physically active if they spend a specific percentage of time inside the facilities. This percentage was chosen based on the assumption that an individual is not physically active 100% of the time inside the facility and taking into account experts’ agreement that members with a history of being previously active in another leisure center were performing physical activities 80% of the time spent inside the facility (losing the other 20% of the time changing clothes, resting between exercises, etc.) (21). With regard to members not being previously active in another leisure center, experts' agreement is that they perform exercise 65% of the time inside the facility, losing about 35% of the time in other endeavors (21). According to these criteria, 67% of members were active enough to be considered for further analysis (*n* = 114,251). Members who were enrolled in a GO fit leisure center but did not attend enough to be considered physically active were excluded from our analysis (33%). Based on the dropout behavior records of members through 15 continuous months, we assumed by experts’ consensus that 80% of GO fit members would continue being physically active during their lifetime.

**Table 1:** Descriptive data of the sample analyzed (*n* = 169,031).

Variable	Individuals (*n*)	Percentage (%)
Gender distribution		
GO fit female members	76,740	45.4
GO fit male members	92,291	54.6
Age groups		
18–29	59,330	35.1
30–44	51,856	30.7
45–59	31,894	18.9
60–69	11,367	6.7
70–79	8,140	4.8
80+	6,444	3.8

### 2.2 Instruments and variables

The PA level of members was estimated using the data of the automatically registered access control by GO fit members. Then, the PIA prevalence in GO fit members (33%) was compared against the Spanish general population PIA prevalence considering gender and age (22).

Following previous studies (21), the method to estimate the burden of PA-related disease involved different steps. First, the most prominent diseases in Spain according to the Spanish National Institute statistics (INE) where PIA is a risk factor were identified, i.e., type 2 diabetes (E11), colorectal cancer (C18, C20, D01.0, and D01.2), breast cancer (C50, C76.1, and C05.9), stroke (I61), and coronary heart disease (I24, I25, and I50; e.g., coronary artery diseases, peripheral artery disease, or kidney problems]. Second, the population attributable fractions (PAFs) for each disease were taken from a previous study (4) [6.2 (1.1–14.2) for type 2 diabetes, 9 (1.7–20) for colon cancer, 8.8 (1.6–19.6) for breast cancer, 5.8 (1–13.1) for stroke, and 5 (0.7–12.1) for coronary heart disease]. Finally, these PAFs [interpreted as the proportion of disease or mortality attributed to a risk factor, PIA in this case (23)] were applied to the number of DALYs in each condition.

### 2.3 Analysis

Lifetime disease burden was calculated using the number of DALYs as the sum of years of life lost (YLL) and years lost due to disability (YLD). Following previous recommendations, an incidence perspective was used to estimate YLLs and a prevalence perspective was used to estimate YLDs (24). Specifically, YLLs were calculated as the number of deaths in 2016 (latest available in INE) multiplied by life expectancy. Model Life Table West level 26 was used for standard life expectancy (men = 80 years; women = 82.5 years) (16). YLDs were calculated as the number of cases multiplied by disability weight multiplied by the duration of the case until remission or death. The different disability weights, taken from Salomon et al. (25), were type 2 diabetes: 0.0116; colorectal cancer: 0.217; breast cancer: 0.0689; stroke: 0.224 (age >60: 0.258); and coronary heart disease: 0.3228. Terminal cancers were considered within mortality not disability. The duration of the diseases was calculated as the difference between the life expectancy and the age at the start of the diseases.

The preferred model was DALY{0;0}, i.e., age weights (*K* = 0) were not included given that DALYs were treated as a measure of population health loss rather than an aspect of social welfare (19) and the discounting rate was 0 (*r* = 0.00) since the study aimed to be a static picture of the burden of disease averted in 2017 and to avoid the inconsistency in the original DALY method regarding the starting time for discounting (i.e., the incidence year in YLD and the year of death in YLL) (26). Nevertheless, sensitivity analyses were conducted to enhance comparability with previous literature. Specifically, the following scenarios were calculated: DALY{*K* = 0; *r* = 0.03}, DALY{*K* = 1; *r* = 0}, and DALY{*K* = 1; *r* = 0.03}. Following Devleesschauwer et al.'s (27) formulae, *K* equals 1 if age weighting is applied and 0 otherwise *β* constant was 0.04 and the constant for age-weighting was C = 0.1658.

The total disease burden of the DALYs was quantified as the DALYs averted by members as a consequence of being physically active. We calculated DALYs for the five diseases mentioned above (i.e., type 2 diabetes, colorectal cancer, breast cancer, stroke, and coronary heart disease) considering gender and age distribution, mortality, and age of deceasing by each age bracket.

## 3 Results

Estimations showed that the activities taking place in GO fit during 2017 could have averted a total of 1,165.74 DALYs (10.96 DALYs per 1,000 members) coming from type 2 diabetes (22.62 DALYs), colorectal cancer (81.16 DALYs), breast cancer (48.72 DALYs), stroke (206.15 DALYs), and coronary heart disease (807.10 DALYs) (Table 2). Most of the DALYs were a consequence of the YLDs potentially prevented in coronary heart disease (770.82 DALYs). By gender, more DALYs were prevented in males (858.16 DALYs) than in females (297.58 DALYs). Specifically, in males, more DALYs per 1,000 members were averted coming from coronary heart-related diseases (11.81 DALYs/1,000), stroke (2 DALYs/1,000), and colorectal cancer (0.83 DALYs/1,000). In females, more DALYs per 1,000 members were averted in breast cancer (0.87 DALYs/1,000) and type 2 diabetes (0.49 DALYs/1,000).

**Table 2:** Total of years of life lost (YLL), years lost due to disability (YLD), and disability adjusted life years (DALYs) averted by GO fit in 2017 as a consequence of delivery of services considering the gender of the members (*n* = 114,251).

Disease	Males				Females				Total			
	YLL	YLD	DALYs	DALYs/1,000	YLL	YLD	DALYs	DALYs/1,000	YLL	YLD	DALYs	DALYs/1,000
Type 2 diabetes	0.18	0.01	0.19	0.00	0.25	22.17	22.42	0.49	0.43	22.18	22.62	0.22
Colorectal cancer	24.20	25.08	49.28	0.83	19.08	12.79	31.87	0.70	43.28	37.87	81.16	0.78
Breast cancer	0.35	0.34	0.68	0.01	24.61	23.42	48.03	0.87	24.96	23.76	48.72	0.43
Stroke	19.40	101.36	120.76	2.05	20.11	65.28	85.39	1.55	39.51	166.64	206.15	1.80
Coronary heart-related diseases	19.20	678.03	697.23	11.81	17.08	92.79	109.87	2.42	36.28	770.82	807.10	7.73

In terms of age (Table 3), the highest general effect of DALYs averted was observed in the 45–59-year age bracket (375.11 DALYs), but the highest DALYs per 1,000 members were observed in the age bracket of 60–69 years’ old (39.51 DALYs per 1,000 members). Considering the gender of the members in the different age groups, a higher number of DALYs were averted in the 45−59-year age bracket in males (300.91 DALYs) and in the 60–69-year age bracket in females (99.75 DALYs). In both cases, the higher number of DALYs averted per 1,000 members was observed in the 60–69-year age bracket (47.86 and 29.24 DALYs averted per 1,000 members in males and females, respectively).

**Table 3:** Total of years of life lost (YLL), years lost due to disability (YLD), and disability adjusted life years (DALYs) adverted by GO fit in 2017 as a consequence of delivery of services considering the age of the members (*n* = 114,251).

Age groups	Males				Females				Total			
	YLL	YLD	DALYs	DALYs/1,000	YLL	YLD	DALYs	DALYs/1,000	YLL	YLD	DALYs	DALYs/1,000
18–29	0.78	140.85	141.63	6.83	0.77	23.25	24.02	1.40	1.55	164.10	165.65	4.58
30–44	3.80	143.96	147.76	8.16	9.98	32.82	42.80	2.50	13.78	176.78	190.56	5.38
45–59	15.37	285.54	300.91	27.01	17.73	56.47	74.20	8.76	33.10	342.01	375.11	20.14
60–69	20.76	169.25	190.00	47.86	34.68	64.67	99.35	29.24	55.44	233.91	289.35	39.51
70–79	17.65	56.22	73.86	25.98	13.41	35.04	48.45	20.78	31.05	91.25	122.31	23.86
80+	4.98	9.02	13.99	6.22	4.57	4.20	8.76	6.15	9.54	13.21	22.76	6.32

Sensitivity analyses showed that the following DALYs that could have been averted considering age-weighting: 886 DALYs {1;0}, discounting: 779 DALYs {0;0.03}, and both: 619 DALYs {1;0.03}.

## 4 Discussion

This study estimated that GO fit could have averted a total of 1,165.74 DALYs during 2017 through the reduction of PIA in its members, or a total of 10.96 DALYs per 1,000 members. Previous analyses have shown that PA interventions have a positive effect on morbidity through the prevention or delay of the onset of chronic diseases larger than the effect on mortality (15), which means that most of the effect is on YLD more than in YLL (17). This was the case in our study, in which 95.5% of the total DALYs came from YLD, with very few deaths prevented. In addition, the reduction of PIA prevalence within GO fit members showed a lower burden of disease per 1,000 individuals in comparison with the general burden of PIA in Spain (4). It is important to recognize that this study focused on GO fit members, which might not completely represent the Spanish population in terms of health behaviors and socioeconomic status. Therefore, future studies should conduct an analysis that includes other fitness centers, to enhance the external validity of the findings.

The disease accounting for the highest number of DALYs potentially averted was coronary heart-related disease (807.10 DALYs averted), followed by stroke-related diseases (206.15 DALYs averted). This is partly consistent with the DALYs averted in a previous analysis considering a PA intervention for transport, in which stroke-related diseases were the most prominent (28). The difference could be explained by the use of different types of disease coding.

With regard to gender, there was a higher number of DALYs averted for males (868.16 DALYs), accounting for 86.4% of the total DALYs averted. This gender difference in the benefits for health can be explained by the differences found in PA involvement, i.e., females exhibit lower attendance frequency and consistency (34) and display a higher prevalence of PIA and lower prevalence of vigorous-intensity activity than males (12). It has been shown that long-term engagement and a higher intensity level provide more benefits for health (35, 36). Therefore, despite the fact that the number of male and female members at leisure centers has been found to be similar (29, 30), the present study shows that centers should keep attempting to close the gender gap. Future studies could explore whether the attractiveness of different fitness center services changes based on gender and the barriers that females face in participation.

By age, the group with the highest DALYs averted was the 45–59-year old one (375.11 DALYs averted), which was also the most prevalent age group in the leisure facilities and the age group with the highest prevalence of the diseases evaluated (30). The 45–59-year old group was also associated with higher YLD averted, while previous studies observed higher effects in YLL (21, 28). This difference requires further research to be explained. When analyzing the effect by 1,000 members, the oldest age groups, 60–69 and 70–79, showed the highest number of DALYs averted (a total of 39.51 and 23.86 DALYs averted by 1,000 members). This is highly relevant given previous studies showing an age gap in which PIA increases with age, while high-intensity exercise decreases in general (12) and more in females (31). Public health policies would, therefore, consider leisure centers potential allies in the improvement of health in older people.

The mixed funding model in which GO fit operates allows private-sector initiatives to contribute to the public health agenda, tackling PIA and thus the burden of disease, while alleviating the burden on public-sector budgets (17). Generally, an allocative inefficiency in society exists in which significant beneficial effects on health at a low expense are not being delivered, while expensive interventions with minimal health benefits are being promoted (32). With this business model, there is no public spending. When the local council is not obtaining an income in the form of an exploitation fee or for renting or selling the land, one would presume that this might be the case, but that is rather difficult in tertiary use of land. As a result of this model, instead of all taxpayers paying for the investment to allow a small group of citizens to be enrolled in leisure centers, just those members enrolled would pay a fixed regulated fee for the services. Usually, the cost-effectiveness threshold for a public health strategy is three times the gross domestic product per person (17). In this model, the gross domestic product per person is zero, while all the risk is supported by the private company. If the gross income of the company is considered, GO fit spends around 27.196,95 Int$ per DALY averted. That money would be considered as a cost-effective strategy and theoretically would be saved by the taxpayers if a direct intervention was implemented. Finally, this model promotes access to PA by disadvantaged groups given its public fixed price. Previous literature shows that the cost of the membership fee in leisure centers might be a barrier for people with lower socioeconomic status (37) and is one of the main reasons to cancel a fitness membership (38). Future studies should include socioeconomic information of participants to explore its role in the effect of PA on health. This is highly relevant when considering that sport contributes to several Sustainable Development Goals (SDGs), including gender equality (SDG 5) and reduced inequality (SDG 10) (33), and the international interest in promoting PA and sport as a social development tool (34).

### 4.1 Limitations

Some limitations of this research should be noted. A fixed number of GO fit members were assumed to continue being active for a lifetime. Nevertheless, the literature recognizes that PA behavior is unsteady (35), which might bias long-term estimations (35). Future research should include dynamic models that account for potential changes in the activity behavior of members to provide a more accurate assessment of long-term health benefits. In addition, the health benefits of the members not reaching the 150min/week recommendation were not considered, but engaging in some PA at the leisure centers is unlikely to be negligible. At the same time, to accurately reflect the effect of physical exercise in health, future studies should include a control group of non-active participants to clearly demonstrate the relative advantages of fitness center services. Also, the cross-sectional design of the study does not allow to establish causality between the levels of PA and the health improvements, which should be addressed in future longitudinal research. Moreover, although it was recognized that only a percentage of the time spent in the facility is dedicated to physical exercise (65%−80%), future studies should use wearables for a more accurate measurement and to consider intensity levels. Finally, some people may not reach the 150min/week threshold in the leisure center but complement it with some activities outdoors. In this case, although a specific attribution analysis should be conducted, leisure centers could act as behavior change facilitators, making members more prone to exercise even outside the facilities. Given these limitations, the present research should be interpreted only as an estimation of the potential role of leisure centers to prevent the burden of disease.

### 4.2 Relevance of this work

To the best of our knowledge, this is first study published with industry data while using the latest estimations of the economic burden of physical inactivity (4). Moreover, it contributes to the understanding of how being active can prevent the burden of different diseases, which is a topic of interest to both the public and the private sectors, given its focus on the contribution of leisure centers to the improvement of public health. Hopefully, the present results will promote the conversation and coordination between academia, private, and civil sectors. Finally, this research supports the evidence that sports, physical activity, and active living help in the development of the United Nations' SDGs (especially SDG 3: good health and wellbeing).

## 5 Conclusion

This study indicates that the estimated reduction in PIA prevalence within GO fit members in 2017 could have averted a total of 1,165.74 DALYs as a consequence of delivery of PA and exercise services, meaning a total of 10.96 DALYs averted per 1,000 members. Because taxpayers do not have to invest in facilities or land, as GO fit provides all the investments and facilities, it seems that this model could become a positive public health intervention that reduces the burden of disease by helping to promote a more active and healthier lifestyle.

## Data Availability

The raw data supporting the conclusions of this article will be made available by the authors, upon request.
